# Introduction: Forgiveness and Conflict

**DOI:** 10.1007/s11406-016-9787-7

**Published:** 2016-12-28

**Authors:** Paula Satne

**Affiliations:** Department of Philosophy, Manchester University, Oxford Road, Manchester, M13 9PL UK

**Keywords:** Personal forgiveness, Conflict, Political forgiveness, Punishment, Reconciliation, Identity, Apologies, Arendt, Kant

## Abstract

The papers collected in this volume are a selection of papers that were presented - or scheduled to be presented - at a workshop entitled Forgiveness and Conflict, which took place from 8-10 September 2014, as part of the Mancept Workshops in Political Theory at the University of Manchester. Some of these contributions are now compiled in this volume. The selected papers draw from different philosophical traditions and conceptual frameworks, addressing many aspects of contemporary philosophical debates on the nature and normativity of forgiveness, including its political aspects. The result is a rich collection of essays which covers a wide variety of philosophical issues, displaying cutting edge scholarship in this area. This introduction provides a brief overview of some of the central themes discussed in the volume with a particular emphasis on their innovative aspects.

The papers collected in this volume are a selection of papers that were presented - or scheduled to be presented - at a workshop entitled *Forgiveness and Conflict,* which took place from 8 to 10 September 2014, as part of the *Mancept Workshops in Political Theory* at the University of Manchester. The workshop consisted of invited and selected papers. Authors were invited to expand on previous published work or present new unpublished papers on the normativity of forgiveness, broadly conceived to include topics related to the nature and definition of forgiveness, its permissibility and its moral, applied, legal and political aspects. Papers that examined the intersection between the moral and political dimensions of forgiveness were particularly welcome.

The workshop included ten papers which draw from different philosophical traditions and conceptual frameworks, generating very lively discussions on many aspects of contemporary philosophical debates on the nature and normativity of forgiveness. Some of these contributions are now compiled in this volume. All papers have been selected after undergoing an ordinary *Philosophia* blind review procedure. The result is a rich collection of essays which addresses a wide variety of philosophical issues, displaying cutting edge scholarship in this area. This introduction provides a brief overview of some of the central themes discussed in the volume with a particular emphasis on their innovative aspects.

In ‘A Plea against Apologies’, Oliver Hallich notes an often neglected *paradox* in the nature of apologies. He argues that apologies involve *attitudinal* elements and thus, a genuinely repentant offender will see the victim’s negative emotions towards him as fully justified. Yet at the same time, apologies also have a *directive* aspect which usually involves a direct or indirect request for forgiveness. Furthermore, the directive aspect of apologies means that by requesting forgiveness, the offender is simultaneously attempting to change the victim’s negative feelings towards him: “ [b]y apologising, the offender tries to bring about a state of affairs which, if genuinely repentant and remorseful, he has no reason to want to bring about” (Hallich 2016). After arguing that different attempts to dissolve the paradox all fail, Hallich arrives at the somewhat surprising conclusion that if we fully and honestly recognise our culpability in relation to our wrongdoing, then we have no rational justification for asking for forgiveness. It turns out that in many cases, not offering our apologies is actually a sign of taking genuine responsibility for our offences, and that often shame is the most morally appropriate attitude towards one’s wrongdoing.

In ‘Forgiveness and Identification’, Geoffrey Scarre also offers an original analysis by focusing on a form of forgiveness that has hardly been discussed (or even identified) in the literature. Scarre notes that most discussions about forgiveness focus on cases in which the victim knows the identity of the offender. Yet, many cases of wrongdoing take place under a veil of anonymity and victims are unaware of the true identities of offenders. Scarre’s contribution makes an attempt to rectify this omission by exploring some of “the conceptual and moral issues for forgiveness that arise in circumstances in which identification of the offender is incomplete, tentative or even incorrect” (Scarre 2016). First, in relation to the conceptual possibility of forgiveness taking place when the victim only has partial information about the offender, Scarre argues that forgiveness is clearly possible, as demonstrated by the example of Gordon Wilson, who granted forgiveness to the unknown member of the IRA who planted the bomb that killed his daughter. But if forgiveness does not require that the victim know the identity of the offender, then there are other possible cases worth examining. For example, a victim might misidentify the offender that she has forgiven. Scarre imagines a case in which some youths are playing football and one of them unintentionally breaks one of your windows. You are not sure which boy was responsible, but you still manage to overcome your anger and forgive the boy under the definite description ‘the boy who broke my window.’ Later, you learn from the account of a neighbour that the boy who broke your window was Craig and conclude that you have forgiven Craig. However, your neighbour is short-sighted and actually the real culprit was Jerry, not Craig. Scarre’s point is that in this case it surely can be said that you have forgiven Jerry, not Craig, because “your forgiveness is targeted via the description ‘the boy who broke my window,’ and that description applies to Jerry and not to Craig” (Scarre 2016). In an interesting variation of this case, it is you who are short-sighted and have misidentified the offender, wrongly believing that you have seen Craig kicking the ball through the window, when in fact the culprit was Jerry. Scarre’s point is that, provided that you would have been equally disposed to forgive Jerry as Craig, then on learning that the real culprit was Jerry, not Craig, you might feel that you did not need to go through the whole process of forgiveness again, because by forgiving Craig, you have already forgiven Jerry *by proxy*. Scarre, then, identifies three conditions for the possibility of this and other forms of ‘forgiveness *by proxy’*. It is important to emphasise that the phenomenon of forgiveness by proxy should not be confused with the more widely discussed phenomenon of ‘*third-party’ or ‘proxy’* forgiveness, that is, cases in which a third party purports to forgive on behalf of another. Scarre argues that forgiveness by proxy does not involve the conceptual or ethical problems usually identified in relation to third-party forgiveness, in particular, the problem of sidelining the original victim. In cases of forgiveness by proxy, it is the victim herself who forgives, not a third-party, hence “*the right persons*- victim and offender- are brought into the relationship of forgiver and forgiven” because “your forgiveness passes through Craig *en route* to Jerry” (Scarre 2016). Scarre ends his paper by considering various other examples in order to clarify under what conditions this type of forgiveness by proxy is possible. The importance of his analysis is that it shows we should not rule out a priori “the idea that one person can ever be the actual recipient of forgiveness mistakenly bestowed on another” (Scarre 2016).

Paula Satne’s contribution, ‘Forgiveness and Moral Development’, addresses the often-neglected topic of Kant’s views on forgiveness. Although Kant does not discuss forgiveness in a lot of detail, Satne argues that there is space in his philosophy for a genuine theory of forgiveness, hoping to lay the grounds for a correct interpretation of this theory. Focusing her analysis on a passage of the *Metaphysics of Morals*, Satne claims that Kantian ethics can ground a wide duty of virtue to be forgiving. She develops a novel argument in support of this duty by drawing on various aspects of Kant’s practical philosophy, including his theory of rational agency, his theories of radical evil and moral development and the formula of humanity. Satne argues against interpretations that see Kant as committed to unconditional forgiveness. Instead, she argues that as an imperfect duty, the duty to be forgiving should be understood as requiring the adoption of a forgiving maxim; that is, as the duty to cultivate a forgiving character; that is, a willingness to forgive wrongdoers under circumstances that are deemed appropriate. According to Satne, considerations related to Kant’s theory of radical evil and moral development make clear that from a Kantian perspective, the chief circumstances that make forgiveness appropriate are repentance of immoral acts as evidence of the wrongdoer’s commitment to a project of self-reflection and self-reform. By appealing to the idea that what Kantian ethics requires is the adoption of a maxim of forgiveness, Satne is able to argue that forgiveness in Kant’s system can include a variety of forgiving attitudes and practices. Since maxims are principles of justification, forgiveness, by Kant’s account, is paradigmatically responsive to reasons and thus particularly well-suited to providing what Hieronymi has called ‘an articulate account of forgiveness’ (2001, p. 530). Finally, Satne claims that Kant’s position also entails that a lack of repentance on the part of the wrongdoer should be taken as evidence of a lack of commitment to a project of moral self-improvement and that, in such cases, Kantian ethics establishes a perfect duty to ourselves not to forgive unrepentant wrongdoers, arguing that this should be understood as one of the duties of self-esteem, which involves the duty to respect and recognise our own dignity as rational and moral beings.

The remaining three papers in the collection examine more closely the possibility of forgiveness and reconciliation in political contexts. Monica Mookherjee’s paper, ‘Healing Multiculturalism: Middle-Ground Liberal Forgiveness in a Diverse Public Realm’, develops a novel conception of *liberal political forgiveness* which is suitable for a multicultural public realm. She argues that this form of liberal forgiveness would constitute a middle ground between two forms of political forgiveness that are ultimately deemed unsuitable for multicultural contexts because they are too extreme. Mookherjee appeals to the case of the Harkis in order to examine the problems and challenges brought about by these different conceptions of political forgiveness, arguing that her moderate account is the best suited to account for these challenges. The Harkis fought as auxiliaries in the French-Algerian war of 1962 and then were seen as traitors by their fellow Algerians, while the French failed to fully support them after the war’s end, thus isolating them from both French and Algerian communities. It has been suggested (Crapanzano 2011, pp. 176–77) that the only solution for the problems currently faced by the Harkis is for them to *forgive* the French. According to Mookherjee, on one side we encounter a conception of political forgiveness, the ‘unattached articulation’ concept, which does not require real emotional change on the forgiver’s part, but rather, a form of civic restraint. The problem with this conception is that it underestimates the “cost to the Harkis and other wounded minorities of having to ‘bracket’ their deepest grievances in the public sphere” (Mookherjee 2016). At the polar opposite end, the ‘change of heart’ approach asks for a strong form of empathy for the perpetrators. Mookherjee argues that this approach is also problematic, insofar as this strong form of empathy is ultimately implausible after deep group conflict. Mookherjee, then, develops her own ‘middle-ground’ conception of political forgiveness by drawing on the notion of ‘compassion for the social world’ and Hannah Arendt’s concepts of natality and ‘love of the world.’ Arendt’s idea of love of the world through the receptivity to newness (‘natality’) supports the rational and emotional shifts that are required for political forgiveness in a diverse public realm, inasmuch as this form of forgiveness does not require that the different groups achieve consensus on a moral judgement about the past without attempting a strong form of empathy or the bracketing of their deepest grievances. Instead, “compassion for the predicament that all would face if forgiveness were absent from the world might motivate the wronged to consider that the other may possess different but reasonable interpretations of history” (Mookherjee 2016). Mookherjee ends her article by developing this middle-ground conception and assessing the various challenges that it faces in relation to different cases of conflict and, in particular, her central case, that of the Harkis.

In ‘Forgiveness, Representative Judgment and Love of the World’, Masa Mrovlje identifies the central political question on the significance and moral appropriateness of forgiveness as the problem of how to acknowledge the seriousness of wrongs committed, while at the same time enabling the possibility of a new beginning that can restore a sense of responsibility for a shared world among former enemies. This question has often been addressed through constructing self-centred, rule-based frameworks, which usually define forgiveness in terms of a moral duty or virtue. According to Mrovlje, the problem with such approaches is that they tend to rely on “a set of prefabricated moral standards” and, as such, they risk abstracting from the historical, situated condition of human political existence and the imperfections that are inherent in it. Mrovlje takes an alternative approach and addresses the central question of political forgiveness from the perspective of Camus’s and Arendt’s aesthetic, worldly, judging sensibility and “its ability to kindle the process of coming to terms with the absurd, and perhaps unforgivable character of reality after evil” (Mrovlje 2016). The worldly perspective of the judging sensibility is oriented by the principle of love of the world and draws on narrative and existential elements in order to inspire a process of reconciliation with the often absurd and always plural character of the world. Mrovlje illuminates this change of perspective by examining two literary accounts of forgiveness in *A Human Being Died That Night* by Pumla Gobodo-Madikizela and *Minuet for Guitar* by Vitomil Zupan. The worldly judgement perspective can foster political forgiveness by “foregrounding it as a situated practice undertaken for the sake of human plurality and the common world” (Mrovlje 2016). Mrovlje is, thus, able to offer a new perspective for thinking about the possibility of forgiveness after deep conflict without ignoring the seriousness of wrongdoing and the suffering that it often brings in its wake. The analysis of two literary works is particularly rewarding for the reader, who is then able to engage imaginatively with the different, but always particular, aspects and perspectives inherent in situations of conflict in order to see forgiveness as opening up the possibility of a new beginning.

In ‘Punishment, Forgiveness and Reconciliation’, Bill Wringe also addresses the issue of how to achieve reconciliation after political conflict, arguing for the interesting and novel conclusion that responding to large-scale violations of human rights via states’ judicial apparatus of trial and punishment does not necessarily undermine the possibility of reconciliation between the conflicting parties. On the contrary, Wringe argues, punishment can sometimes be the vehicle for political reconciliation. Wringe examines various attempts in the literature to dissolve the apparent tension between punishment and reconciliation arguing that they all fail for a variety of reasons and in particular because they do not provide a plausible account of political reconciliation. In order to dissolve the tension, Wringe claims that what is needed is a “more careful consideration of what is constitutively involved in both punishment and political reconciliation” (Wringe 2016). He provides the required analysis by appealing to the ‘denunciatory expressivism’ view of punishment, a view defended in recent published work (Wringe 2016a, b) and to Moellendorff’s notion of political regret (Moellendorff 2007) according to which the expression of regret is partly constitutive of political reconciliation. Political regret involves the *public* acknowledgment and repudiation of past wrongdoing, that is, an acknowledgment which is made *on behalf of* the public *to the public*. On the denunciatory view, the intended audience of punishment is the members of the political community whose rules have been infringed, as such punishment can be the vehicle for the public expression of political regret for state-sponsored wrongdoing, and thus it can be partly constitutive of political reconciliation. On this view, punishment of state-sponsored wrongdoing is the pre-eminent means by which political regret should and can be expressed. It follows that punishment and reconciliation are not only not necessarily in tension, but also that punishment might be required for reconciliation in a way that makes the easy resort to amnesty politically undesirable.

Each paper presents its own approach and develops its own independent line of argument, but I wish to end this short introduction by providing a quick overview of some areas of intersection, agreement and disagreement between the different contributions included in this volume. One common issue, discussed explicitly or implicitly by some of the authors, is the question of whether the wrongdoer’s repentance or change of heart provides a reason for forgiveness. Hallich’s analysis relies on the view that forgiveness has a gift-like nature and that, therefore, there could not be a duty to forgive wrongdoers: “forgiveness is a free gift which the victim may grant or withold without being irrational…she never has a duty to forgive” (Hallich 2016). Hallich argues that the offender’s remorse and repentance do not provide *reasons* for the victim to forgive the wrongdoer and, in particular, that they do not provide reasons for the wrongdoer to apologise as a way of demanding forgiveness in light of his repentance (Hallich 2016). Forgiveness is, thus, not morally or rationally required. In contrast, Satne’s analysis entails that, at least from a Kantian perspective, there is a duty to be forgiving, understood as a duty of virtue that involves the cultivation of a forgiving character through the adoption of a maxim of forgiving others in circumstances that are deemed appropriate. According to Satne’s reconstruction of Kant’s position, the appropriate circumstances for forgiveness are chiefly the repentance of immoral acts as evidence of the wrongdoer’s commitment to a project of moral reformation and this, of course, implies that by Kant’s analysis, repentance *often* provides a reason to forgive wrongdoers (Satne 2016). Yet the disagreement between Satne and Hallich is not as deep as it might seem at first glance. The Kantian duty to be forgiving is a wide duty of virtue; as such, it does not correspond to a *right* of the wrongdoer to be forgiven. To this extent, Satne can agree with Hallich that offering an apology as a form of requesting, or even demanding, forgiveness is not morally appropriate. It is worth pointing out that Scarre’s contribution might also have some implications for this debate as his analysis seems to imply that repentance is not always a necessary condition for the moral appropriateness of forgiveness. If forgiveness is both possible and appropriate in cases in which the forgiver has very little information about the offender, and if forgiveness by proxy is a real possibility, then we might expect that in many cases, the forgiver would not know whether or not the offender has repented. Thus, Scarre’s analysis ultimately implies that repentance in many cases is not a necessary condition for the moral appropriateness of forgiveness. However, Scarre also allows for the possibility of forgiving an unknown offender while knowing that he is remorseful because of the testimony of a third party and he is keen to point out that in most cases, forgiveness is unlikely to achieve reconciliation where an offender is unrepentant (Scarre 2016, note 1).

The contributions by Mookherjee and Mrovlje overlap in many interesting ways. Both authors appeal to Hannah Arendt’s conception of love of the world as a way of conceptualising forgiveness, which is preferable to some competing accounts which are seen as problematic. However, there are also some differences in their approaches. In the case of Mookherjee, the appeal to Arendt’s conception of natality and love of the world is taken to provide the grounds for a moderate conception of political forgiveness which is both liberal and particularly well-suited to dealing with the challenges that forgiveness possesses in multicultural societies in which there are competing interpretations of the past. In contrast, Mrovlje appeals to Arendt’s conception (and Camus’s existentialism) in order to offer an alternative account to the prefabricated moral standards that are imposed onto the particularities of the political world from the outside and above and that are usually preferred in contemporary transitional justice literature. To the extent that it does not require forgiveness as a means to achieve political reconciliation in cases of large-scale state-sponsored wrongdoing, Wringe’s approach contrasts with both Mrovlje’s and Mookherjee’s approaches. On one hand, political reconciliation does not demand that each individual citizen becomes reconciled with each of their fellows, and thus, it does not require that each victim forgives the wrongdoers. Such requirement would be too demanding and in certain cases it might also be politically inappropriate or even morally problematic. On the other hand, the possibility that political reconciliation involves the idea of groups forgiving one another is not plausible because in many cases “reconciliation (if it takes place) will be a relationship between relatively unstructured groups, such as ethnic and cultural groups, which do not meet the conditions for collective agency” (Wringe 2016). Instead, the acceptance of wrongdoing that is required for political reconciliation might be achieved through state- actions, more specifically, through the expression of political regret through punishment of state-sponsored wrongdoing, understood in a suitable denunciatory way.

Here, I have only been able to provide a brief summary of the main lines of arguments developed in the different contributions and some of the points of agreement and disagreement among the authors but of course, the arguments developed in the different papers are far more complex and richer than this brief introduction can justify. Forgiveness as a positive response to wrongdoing plays a role in the moral lives of most ordinary human agents and it is even more significant in the wake of deep political and social conflict when wrongdoing has been widespread and divisive. This volume invites readers to reflect on the nature and moral appropriateness of personal and political forgiveness; it would be equally attractive for specialists who are searching for new developments in this area but it would also be appealing to those who are new to the topic because it discusses a wide variety of issues from different perspectives and philosophical traditions.
